# Bone Fracture Patterns and Distributions according to Trauma Energy

**DOI:** 10.1155/2022/8695916

**Published:** 2022-09-09

**Authors:** Ahmad Almigdad, Ayman Mustafa, Sattam Alazaydeh, Mu'men Alshawish, Mohammad Bani Mustafa, Hamza Alfukaha

**Affiliations:** Department of Orthopedic, Royal Medical Services, Amman, Jordan

## Abstract

**Background:**

This study investigates the effect of injury mechanism and energy on fracture patterns and distributions. Also, it compares differences in bone fracture patterns based on injury mechanism, gender, and age.

**Methods:**

Three thousand and sixty-six admitted patients with bone fractures were reviewed retrospectively, and the fractures were analyzed regarding age, gender, and mechanism of injury. Fractures were located in eleven bones. However, the forearm, hand, leg, and foot were considered one bone, and the fracture was then subclassified according to the anatomic position within each bone. Trauma energy was classified according to the mechanism of injury where simple falls were considered low-energy injury while falling from a height, road traffic accidents, bullet, and industrial injuries were considered high energy.

**Results:**

Males represented most of the patients, and most injuries occurred in adults. However, the male patients were more prone to injuries than females across all age groups below fifty years, women above fifty years were more frequent, and a third of females' injuries occurred in the elderly. Simple falls represent two-thirds of the trauma mechanism, and falling from a height and road traffic accidents are the most common high-energy injuries and more prevalent in males. Scapular, clavicular, distal humerus, and shaft of long bones fractures were more prevalent in males. In contrast, females had a higher frequency of proximal humerus, proximal and distal femur, distal leg, and thoracic spine fractures. Industrial injuries are more frequent in males; thus, hand injuries are more frequent. Pathological fractures were higher in females, and spine and pelvic fractures were more associated with high-energy injuries.

**Conclusions:**

The trauma's energy determines the bone injury's extent and nature. Knowing the trauma mechanism is essential to expect the extent of injuries and construct preventive measures accordingly.

## 1. Background

Trauma is the leading cause of death in individuals aged 1 to 44 [1]. In high-income countries, road traffic injuries and self-inflicted and violent injuries are the leading cause of death in people 15–29 years old, while 75% of deaths occur in men [2]. The majority of injuries are preventable. However, ninety percent of trauma-related deaths occur in low- and middle-income countries, secondary to many factors such as lack of prevention and immediate and quality trauma care [3].

Traumatic events transfer the energy to the body from an outside force, and increasing injury force increases the energy that transfers to the body and results in more body damage. Trauma may be blunt or penetrating in nature. Some trauma combines multiple injuries; for example, blast and mass casualty events may have combined blunt and penetrating injuries and thermal and chemical injuries [4, 5].

Trauma frequently causes bone injuries and contributes to significant mortality and morbidity. Fracture patterns and associated injuries are attributed to many factors, such as the age of patients, bone quality, and pre-existing bone pathology. However, the mechanism of injury is the predisposing and most important factor for injury. The kinetics of energy, patient position, and available preventive measures affect the force that transfers to a certain body part and the consequent injury pattern [6–10].

This review investigates the effect of injury mechanisms and energy on fracture patterns and distributions and also compares differences in bone fracture patterns based on injury mechanism, gender, and age.

## 2. Methods

This retrospective study reviewed the clinical and radiological records of all patients admitted with bone fractures to the Royal Rehabilitation Center (RRC) at King Hussein Medical City (KHMC) in Amman, capital of Jordan, from July 2018 to December 2021.

In this study, we include (a) all RRC inpatients admitted with bone fracture from all age groups from July 2018 to December 2021, (b) patients who have a clear cause of the mechanism of injury, and (c) patients who had available radiographs on Picture Archiving and Communication System. We excluded (a) all patients who were discharged from the emergency department because their records were lacking, (b) patients who were readmitted for reoperation due to failure, infection, and nonunion, and (c) patients whose mechanism of injury is not documented or clear in their records. Therefore, we exclude mechanisms such as sports injury and violence. We found that 3066 records out of 3297 met the inclusion criteria and were enrolled in our study.

Sociodemographic and clinical data were extracted from patients' records, and their radiographs were reviewed to analyze fracture locations and patterns. The patients' age, gender, mechanism of injury, type of fracture, and associated injuries were obtained. Age groups were categorized as follows: children (≤9 years), adolescents (10–18 years), adults (19–70 years), where each decade represented a category till 70 years old, and elderly patients (≥71 years). Falling from height, road traffic accidents, and bullet and industrial injuries were considered high-energy injuries, while simple falls and falling from ground level were considered low-energy injuries.

Fractures were classified into eleven bones where forearm, leg, hand, and foot were considered one bone each. Each bone is classified according to the anatomical location into proximal, shaft, and distal for long bones and the spine into cervical, thoracic, and lumber for spine fracture. However, sacral and coccygeal fractures were allocated with pelvic fractures because they are mostly associated with pelvic injuries. Hand and foot were categorized into carpal and tarsal, metacarpal, metatarsal, and phalangeal. Associated injuries such as neurovascular and open injuries were analyzed with each fracture pattern.

### 2.1. Statistical Data Analysis

The mean and standard deviation were used to describe the continuously measured variables, and the frequency and percentages were used to describe the categorically measured variables. The Kolmogorov–Smirnov statistical normality test and the histograms were used to assess the statistical normality assumption for the measured parameters. The chi-squared test of independence was used to assess the correlations between categorically measured variables. Continuity-corrected and Likelihood Ratio corrected chi-squared tests of independence were used where statistical assumptions were violated for 2^*∗*^2 or higher contingency tables, respectively.

The independent samples *t*-test was used to assess the statistical mean differences in metric variables across the levels of binary categorical variables. The One-way ANOVA test was used to assess the statistical mean differences in metric variables across the levels of more than two categorical measured variables. Welch's adjusted ANOVA was employed for One-way ANOVA tests with unequal variances.

The Multivariate Binary Logistic Regression was used to assess the statistical significance of the impact of key relevant measured independent predictor variables on patients' odds of presenting with complicated and severe bone injury upon presenting to the hospital, and the association between the tested factors with the analyzed outcomes was expressed as an Odds Ratios (OR) with their associated 95% confidence intervals. The SPSS IBM V21 statistical data analysis program was used for the data analysis. The alpha significance level was considered at 0.050 level.

## 3. Results

**Figure 1:**
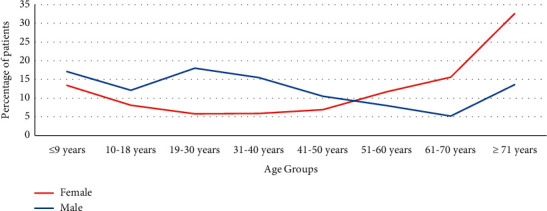
Comparison between males and females' bone injury frequency across age groups.

Three thousand and sixty-six medical records for patients admitted to the Royal Rehabilitation Center with bone fractures were reviewed retrospectively. Males represented most of the patients (59.1%). The mean age for the patients was equal to 42.15 ± 26.69 years. However, the mean age for male patients (36.04 ± 24.45) was younger than for females (51.06 ± 27.61). The majority of the patients were in the adult group, representing 52.5%. Children and adolescents represented 15.6% and 10.5%, respectively, while the elderly formed 21.4% of the sample. However, the male patients were significantly more prone to injuries than females across all age groups below fifty years, but women above fifty years were significantly more prone to injuries than males. Around a third of females' injuries occurred in the elderly (Table 1; Figure 1).

Most injuries were low-energy injuries caused by simple falls, representing 68.6%. The most common mechanism for high-energy injuries was falls from height, representing 16.5%, followed by road traffic accidents, which accounted for 12.7%. However, males were more prone to high-energy fractures within all injury mechanisms than females.

Most fractures occurred in the lower limb (45.4%), followed by upper limb fractures (33.4). Spine and pelvic fractures accounted for 16.2% and 5%, respectively. Long bone fractures and spine were the most common cause of orthopedic hospitalization, and the femur accounted for around a quarter of admissions (24.1%). The proximal femur, distal humerus, lumbar spine, and distal leg were the leading causes of hospitalizations. However, hand and foot accounted for 3.7% and 4.8% of admissions. The scapula (0.3%) and the patella (0.8%) were the least frequent fractures (Table 2). Male patients were significantly more predicted for scapular, clavicular, distal humerus, the shaft of the long bones, and hand phalangeal and tarsal bone fractures. On the other hand, the female patients were significantly more predicted for the proximal humerus, proximal and distal femur, distal leg, and thoracic spine fractures.

Compound fractures at presentation accounted for 5.97%, and 1.57% of fractures were associated with vascular injuries that mandated vascular surgeon interventions. However, pathology was found in 3.1%, and neurological injuries at presentation accounted for 2.1%. Male patients were also more significantly predicted for associated neurovascular injuries and compound fractures. In contrast, pathological fractures were more frequent in female patients (Table 3).

**Figure 2:**
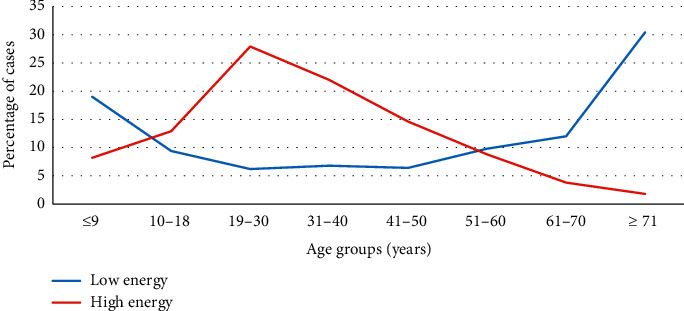
Comparison between high- versus low-energy injuries across age groups.

Table 4 revealed the association between patients' age and associated injuries with injury mechanisms. When comparing the mean age according to the different mechanisms of injury, there were meaningful differences. The patients' injuries caused by simple falls were the most responsible mechanism in elderly patients and children. However, high-energy mechanisms were blamed on younger patients. Patients aged 19–30 years were significantly more predicted for falls from height, gunshots, and RTA, but people aged 41–50 years were also significantly more predicted to have industrial injuries (Figure 2). The associated neurovascular and compound fractures were significantly more predominant with gunshots, industrial, and RTA's injuries. Nevertheless, pathological fractures were caused mainly by simple falls.

Additionally, the affected body region correlated significantly with the mechanism of injury; pelvic fractures were more predominant in RTA, while spine fractures were more significant with falls from height and RTAs. Gunshot injuries predominantly affected the lower limbs, while industrial injuries were frequently noticed in upper limb injuries, particularly hand fractures. Left limbs were injured more frequently with gunshot mechanisms and simple falls. Yet, axial body fractures were more predicted from falls from height.

Scapular and clavicular fractures were significantly more prevalent among patients' injuries caused by falls from height and RTAs. Most distal humerus fractures were caused by the low-energy mechanism, simple falls. However, proximal and distal femur were mostly caused by simple falls, while femoral shaft fractures were more prevalent with RTA's mechanism. Similarly, proximal leg and leg shaft fractures were significantly more predicted among patients with falls from height, RTAs, and gunshots. In contrast, distal leg fractures were caused mainly by simple falls (Table 5).

**Figure 3:**
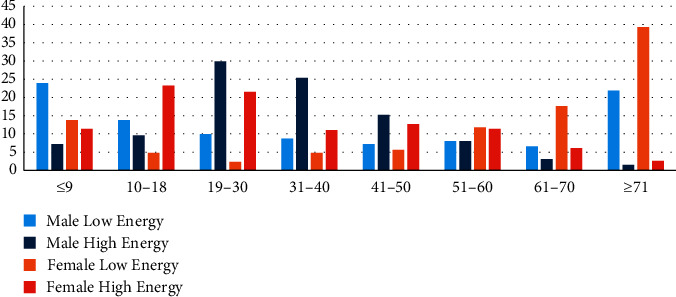
Fracture frequency according to injury energy across age groups.

Injury energy was analyzed within the same gender to compare the difference between high and low energy. Patients who endured high-energy injuries were significantly younger than those who endured low-energy injuries in both genders. More than two-thirds of high-energy injuries in males were located within 19–50 years. Similarly, female patients aged 10–30 years were more predicted to have high-energy injuries (Figure 3; Table 6).

Fracture patterns were analyzed within gender; fractures were compared within the low- and high-energy categories separately and emphasized the previous results (Table 7). Males in low-energy injuries were significantly younger than females; on the other hand, in high-energy injuries, there was no difference in mean age between both genders, and the patients were younger than the low-energy group.

**Figure 4:**
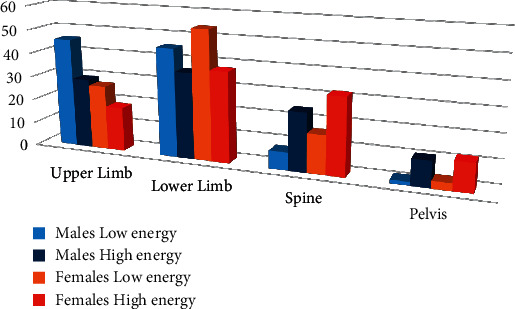
Fracture frequency according to injury energy according to the anatomical region of the body.

**Figure 5:**
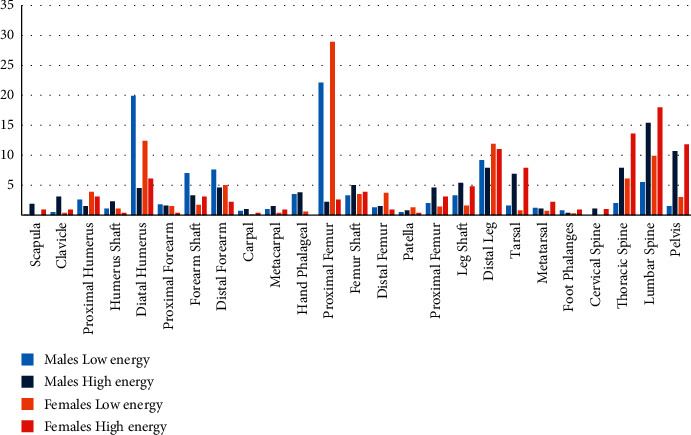
Fractures distributions according to gender and injury energy.

Also, upper and lower limbs were affected equally in males, while the lower limb fractures were more predominant in females (Figure 4). Male patients were significantly more predicted for the distal humerus, forearm shaft, and hand phalanges. However, females with low-energy injuries were more prone to proximal and distal femur and spine fractures than males (Figure 5). Pathological fractures were more common in females, and open fractures were commoner in males.

**Figure 6:**
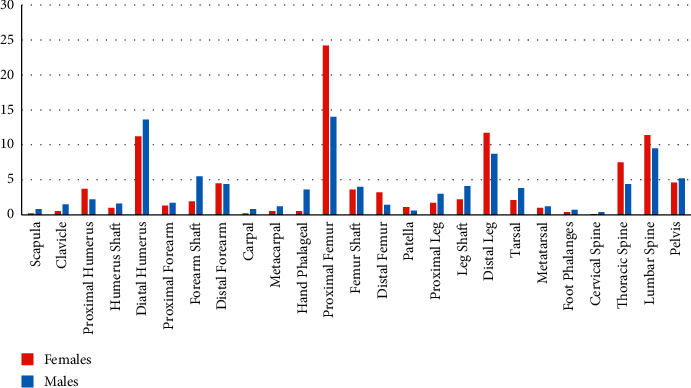
Fractures distributions according to gender.

Fracture patterns in high-energy injuries were comparable in both genders. However, males had a higher frequency of hand phalangeal fractures, while females had higher thoracic spine fractures. Furthermore, fracture-associated injuries, mainly compound and vascular injuries, were more frequent in males (Figure 6).

Forty-seven patients were admitted with joint dislocation. Hip dislocation was the most frequent (19 patients with a frequency of 40.4%); two patients had associated sciatic nerve palsy. Ankle dislocation was the second most frequent (10 patients, 21.3%), and four had open dislocation. Elbow dislocation was the third joint dislocation (8 patients, 17%), and three had an open dislocation (Table 8).

## 4. Discussion

In trauma, physics principles are essential in determining injuries. The load velocity applied to the body determines damage (force = mass × acceleration). If the object causing the trauma is deformable, the time to impact increases, and consequently, the damage increases. In case both objects move, such as in motor vehicle collisions, kinetic energy transferred is additive, 1/2 mass × velocity^2^ [11–14].

In motor vehicle collisions, injury patterns vary according to the side of impact. Frontal impact collisions can cause knee, thigh, and hip injuries. Position of femur weather adducted and abducted affects the force vector and determines the acetabular fracture pattern and associated hip dislocation. Side impact collisions may result in pelvic fracture [15]. Additionally, spine fracture occurs especially in the cervical region with acceleration-deceleration injuries, which cause whiplash-associated disorders [16, 17]. Motorcycle crashes may result in open-book pelvic fracture secondary to the pelvis striking the handlebars.

Injuries to a pedestrian struck by a vehicle depend on the age and height of the individual. Short adults may develop femur fractures, while taller individuals may develop tibia fractures. The victim may be thrown away, causing extremity, pelvic, head, and neck injuries. Larger vehicles strike at a higher site causing injuries to the pelvis, abdomen, or chest [18–20].

Falls are more prevalent in children and the elderly. Injuries are determined by the mechanism of fall and landing position. Intentional falls mandated psychiatric support as well. Simple falls from the ground level affect mainly elderly individuals secondary to bone fragility, where hip fractures are frequent injuries. Falling from height leads to axial loading and causes severe injuries, and if the victim landed on the feet, this may result in calcaneus and long bone and spine fractures [21–23].

Penetrating injuries cause injuries along the pathway of the object. Injuries are determined by the object's velocity, size, and location of the injury. Bullets cause injuries not just by penetrating the body; they also cause thermal, shock waves, and cavitation injuries, especially with high-velocity weapons. The path of the bullet is not necessarily straight, and therefore the path of the bullet needs exploration. Stabbing injuries are low-velocity injuries, and the injury occurs along the path of entry. Penetrating injuries result in open injuries with direct communication of underlying injuries with the external environment, which predisposes to infection [24, 25].

Falls are the leading cause of emergency department visits and admissions [26]. Trauma remains the leading cause of death for children over one year and the second cause for children under ten years. Motor vehicle collision is the leading cause of injuries in children. A child's body does not fit the seat belt; therefore, a car seat is essential for a child younger than eight years. Toddlers are prone to falls secondary to awkward gait and large heads [27, 28].

Bergh et al. studied 27,169 fractures over four years in the catchment area in Sweden. The five most common fractures accounted for more than 50% of all fractures: distal radius, proximal femur, ankle, proximal humerus, and metacarpal fractures. 9.2% of the registered fractures were caused by high-energy trauma, and the proportions of open fractures were 2.3% for all fractures [29]. Because Bergh includes all the fractures in the Swedish Fracture Register for patients older than 16 years, this explains the difference in values in our study, as we assessed hospitalized fractures in all age groups. And many fractures in the distal radius, proximal humerus, and metacarpal fractures were discharged from the trauma unit and not calculated. High-energy fractures in our study represented 31.4% of all injury mechanisms because most discharge fractures are believed to be due to low energy. Therefore, the high-energy injuries represented a high percentage in the study sample. The incidence of open fracture in our study is higher (5.97%) for the same reason.

Traffic accidents are a leading cause of hospitalizations for injuries worldwide [30]. In addition, according to the World Health Report 2010, traffic accidents were identified as the ninth leading cause of disability for all age and gender categories. Most mortality from RTA occurs in developing countries due to a lack of safety measures [31].

However, in our review, road traffic accidents accounted for 11.5% of hospitalizations for fractures, a much lower than the actual incidence since only isolated bone injuries are admitted to the orthopedic department of our institute. In contrast, those associated with other injuries and multiple fractures are often admitted to trauma and surgical wards and not counted. Furthermore, grouping all traffic accidents into a single category prevents their analysis and the construction of preventive measures to minimize these injuries.

Airaksinena et al. analyzed data from patients with severe traffic accidents aged ≥16 years from the Helsinki Trauma Registry in Finland covering the years 2009–2018; 38.6% were occupants of motor vehicles, 28.5% were motorcyclists or moped riders, 17.2% were cyclists, and 15.7% pedestrians. Seriously injured pedestrians and cyclists were older and had a higher mortality rate than motorcyclists and motor vehicle occupants. Overall injury severity was the highest among pedestrians, followed by cyclists [32]. Mansuri et al. conducted a systematic review of all articles published on road accidents in Saudi Arabia over the last 25 years. Traffic accidents are the leading cause of admissions due to injuries, with the male and young age groups being the most affected. Excessive speeding was the most common cause of RTA [33].

Fractures are associated with morbidities other than musculoskeletal sequelae. For example, pelvic fractures are high-energy injuries associated with sexual dysfunction. Rovere et al. conducted a systematic review to assess sexual dysfunction associated with pelvic ring injuries and found that the incidence of sexual dysfunction was related to the type of pelvic ring fracture. Patients with anterior and posterior compression-type and vertical shear-type fracture patterns reported a higher incidence and severity of sexual dysfunction. However, only a weak association was found between genitourinary injuries, the occurrence and severity of sexual dysfunction, and the association between surgical treatment and sexual dysfunction [34].

Our review aimed to study fracture patterns and distributions based on injury energy between genders and age groups. However, we included patients admitted to orthopedic wards with bone fractures needing surgical fixation or observation, while patients discharged from the emergency department or treated as outpatients were not counted. Therefore, the actual incidence of all fractures was not evaluated, and those outpatient-treated fractures are believed to be secondary to low-energy injury and not included in the analysis. Accordingly, our analysis might be biased toward high-energy injuries. However, we believe that this analysis is important to better understand the effect of injury mechanisms on fracture patterns and distributions within different age groups and genders.

Males represented the majority of admitted patients with bone fractures (59.1%). The mean age for male patients is lower than for females in low-energy injuries. However, there were no gender differences in the mean age in high-energy injuries; this may be explained that, in high-energy injuries, the main factor determining the fracture patterns and distribution is the mechanism of injuries, while in the case of low-energy injuries, bone quality plays an important factor. However, women are more prone to osteoporosis than men and at higher risk for fragility fractures [35, 36]. Males had a higher prevalence of high-energy injuries from all mechanisms; falling from height and road traffic accidents were the most frequent. The upper and lower limbs were affected equally in low-energy injuries, while the lower limbs were more frequent in high-energy injuries. Compound fractures and neurovascular injuries were higher in males, secondary to higher high-energy injuries. Comparably, high-energy mechanisms blamed for scapular, clavicular, distal humerus, and the shaft of the femur, leg, and humerus fractures were more prevalent. Industrial injuries are more frequent in males, and thus, hand injuries are more frequent. Tarsal fractures were more frequent in males too.

Females older than fifty years were more prone to fractures than males, and more than two-thirds of admitted female patients were older than 50 years. More than 80% of injuries in females were caused by simple falls, and the prevalence of pathological fractures was higher in females. Females were less likely to be exposed to high-energy injuries because they were less likely to work in manual labor and industrial work and less likely to be involved in road traffic accidents. Lower limbs were more likely to be injured in females, and proximal humerus, proximal and distal femur, distal leg, and thoracic spine injuries were the most frequent injuries.

Simple falls were the most common injury mechanism. However, the actual prevalence is expected to be higher because patients treated as outpatients were not included. Lower limbs were more frequent than upper limbs, and simple falls were the most blamed mechanism in children and elderly patients. Low-energy mechanisms cause fractures in the distal humerus, proximal and distal femur, and distal leg.

Falls from height were the most common high-energy injury mechanism, followed by road traffic accidents. Male are more likely to be injured because they are more prone to road traffic accidents and are exposed to manual labor and industrial injuries. Spine and pelvic fractures are high-energy injuries caused mainly by falling from height and road traffic accidents. Scapula and long bone fractures were caused mainly by high-energy injuries. Cervical spine injuries are high-energy injuries. However, the prevalence of cervical fractures was not represented accurately in our study because cervical spine fractures in our institute are admitted to the neurosurgery department. Those counted in our study were associated with other injuries and admitted to the orthopedic department.

Similarly, joint dislocations in our review represented 1.5% of all admitted patients; this is lower than expected because most cases were treated as outpatients and not counted. In a regional review from Tehran by Nabian et al., joint dislocation represented 3.3% of musculoskeletal injuries [37].

In 2020, the world took strict measures to enforce social distancing to contain the spread of the COVID-19 virus. These include transport restrictions, working from home, and the closure of industries and many facilities. Therefore, this is reflected in the decrease in injuries in general, mainly traffic and industrial injuries. In 2021, trauma admissions increased mainly due to traffic accidents as many COVID-19-related restrictions were lifted. Al Rousan et al. conducted a study at our institute to measure the impact of social distancing on geriatric hip fractures in the Jordanian population during the COVID-19 pandemic. A decrease in the total number of trauma patients and an increase in geriatric hip fractures were noted due to the inability of caregivers to reach the geriatric relative. Therefore, older people are forced to be more dependent, which increases the risk of falling [38]. We are conducting a comparative study to assess the effect of COVID-19 on fracture patterns and mechanisms during COVID-19 and the year preceding and following. However, we are awaiting the data for 2022.

### 4.1. Limitation of the Study

The retrospective design and the lack of adequate documentation of the mechanism of injury preclude a detailed analysis of the etiology. Grouping the mechanism of injury into broad categories, such as simple falls and falls from a height, makes it difficult to identify the exact causes of the injury and establish future preventive measures. Furthermore, classifying bone into regional anatomies prevents detailed analysis of each fracture alone.

This study included patients hospitalized in orthopedic units, while patients discharged from the emergency department or treated as outpatients were not counted. Therefore, the actual incidence of all fractures was not evaluated, and those outpatient-treated fractures are believed to be secondary to low-energy injury and not included in the analysis. Consequently, our analysis could be biased toward high-energy injuries.

## 5. Conclusions

Many factors contribute to musculoskeletal injury patterns; these include patients' age, bone quality, and injury mechanism. However, the trauma's energy determines the bone injury's extent and nature. Simple falls are the most common injury mechanism in both genders, while males are more prone to high-energy fractures. Each injury mechanism contributes to specific fracture patterns in each gender. Therefore, knowing the trauma mechanism is essential to expect the extent of injuries and plan preventive measures accordingly.

## Figures and Tables

**Table 1:** Bivariate comparison between male and female bone injury patients, *n* = 3066.

	Total	Female	Male	Test statistic	*p*-value
Frequency	3066 (100)	1254 (40.9)	1812 (59.1)		

Age (years), mean (SD)	42.15 (26.69)	51.06 (27.61)	36.04 (24.45)	*t* (3046) = 15.85	<0.001

Age groups					
Children (≤9 years)	478 (15.6)	168 (13.4)	310 (17.1)	*χ*2(7) = 391.9	<0.001
Adolescent (10–18 years)	321 (10.5)	102 (8.1)	219 (12.1)		
Adult	1611 (52.5)	575 (45.9)	1036 (57.2)		
19–30 years	399 (13)	73 (5.8)	326 (18)		
31–40 years	355 (11.6)	74 (5.9)	281 (15.5)		
41–50 years	276 (9)	86 (6.9)	190 (10.5)		
51–60 years	292 (9.5)	147 (11.7)	145 (8)		
61–70 years	289 (9.4)	195 (15.6)	94 (5.2)		
Elderly (≥71 years)	656 (21.4)	409 (32.6)	247 (13.6)		

Fracture injury energy					
Low energy	2102 (68.6)	1026 (81.8)	1076 (59.4)	*χ*2(1) = 173.1	<0.001
High energy	964 (31.4)	228 (18.2)	736 (40.6)		

Mechanism of fracture					
Falls from height	507 (16.5)	151 (12)	356 (19.6)	*χ*2(4) = 196.40	<0.001
Gunshot	30 (1)	2 (0.2)	28 (1.5)		
Industrial	38 (1.2)	0	38 (2.1)		
RTA	389 (12.7)	75 (6)	314 (17.3)		
Simple falls	2102 (68.6)	1026 (81.8)	1076 (59.4)		

**Table 2:** Bivariate comparison between male and female bone injury patients.

	Total	Female	Male	Test statistic	*p*-value
Upper limb	1024 (33.4)	318 (25.4)	706 (39)		

Scapula	17 (0.3)	2 (0.2)	15 (0.8)	*χ* ^2^(1) = 6.03	0.014

Clavicle	34 (1.1)	6 (0.5)	28 (1.5)	*χ* ^2^(1) = 7.69	0.006

Humerus	515 (16.8)	200 (15.4)	315 (17.4)		
Proximal	86 (2.8)	47 (3.7)	39 (2.2)	*χ* ^2^(1) = 6.92	0.009
Shaft	41 (1.3)	12 (1)	29 (1.6)	*χ* ^2^(1) = 2.33	0.127
Distal	388 (12.7)	141 (11.2)	247 (13.6)	*χ* ^2^(1) = 3.82	0.051

Forearm	342 (10.6)	96 (7.7)	246 (13.8)		
Proximal	47 (1.5)	16 (1.3)	31 (1.7)	*χ* ^2^(1) = 0.929	0.335
Shaft	123 (4)	24 (1.9)	99 (5.5)	*χ* ^2^(1) = 24.30	<0.001
Distal	172 (5.6)	56 (4.5)	116 (6.4)	*χ* ^2^(1) = 5.25	0.022

Hand	116 (3.7)	14 (1.1)	102 (5.6)		
Carpal	16 (0.5)	2 (0.2)	14 (0.8)	*χ* ^2^(1) = 5.37	0.021
Metacarpal	28 (0.9)	6 (0.5)	22 (1.2)	*χ* ^2^(1) = 4.43	0.035
Phalangeal	72 (2.3)	6 (0.5)	66 (3.6)	*χ* ^2^(1) = 32.4	<0.001

Lower limb	1391 (45.4)	640 (51)	751 (41.4)		

Femur	740 (24.1)	388 (30.9)	352 (19.4)		
Proximal	557 (18.2)	303 (24.2)	254 (14)	*χ* ^2^(1) = 51.4	<0.001
Shaft	118 (3.8)	45 (3.6)	73 (4)	*χ* ^2^(1) = 0.39	0.533
Distal	65 (2.1)	40 (3.2)	25 (1.4)	*χ* ^2^(1) = 11.72	0.001

Patellar	25 (0.8)	14 (1.1)	11 (0.6)	*χ* ^2^(1) = 2.37	0.123

Leg	482 (15.7)	195 (15.6)	287 (15.8)		
Proximal	76 (2.5)	21 (1.7)	55 (3)	*χ* ^2^(1) = 5.68	0.017
Shaft	102 (3.3)	27 (2.2)	75 (4.1)	*χ* ^2^(1) = 9.10	0.003
Distal	304 (9.9)	147 (11.7)	157 (8.7)	*χ* ^2^(1) = 7.76	0.005

Foot	144 (4.8)	43 (3.4)	101 (5.6)		
Tarsal	94 (3.1)	26 (2.1)	68 (3.8)	*χ* ^2^(1) = 7.03	0.008
Metatarsal	33 (1.1)	12 (1)	21 (1.2)	*χ* ^2^(1) = 0.30	0.594
Phalangeal	17 (0.6)	5 (0.4)	12 (0.7)	*χ* ^2^(1) = 0.923	0.334

Spine	498 (16.2)	238 (19)	260 (14.3)		
Cervical	9 (0.3)	1 (0.1)	8 (0.4)	*χ* ^2^(1) = 3.314	0.069
Thoracic	174 (5.7)	94 (7.5)	80 (4.4)	*χ* ^2^(1) = 13.14	<0.001
Lumbar	315 (10.3)	143 (11.4)	172 (9.5)	*χ* ^2^(1) = 2.93	0.087

Pelvis	153 (5)	58 (4.6)	95 (5.2)	*χ* ^2^(1) = 0.59	0.44

**Table 3:** Fracture-associated injuries at the time of presentation, *n* = 347.

Injury	Frequency	Females	Males	Test statistic	*p*-value
Open fracture	183 (5.97%)	24 (1.9)	159 (8.8)	*χ* ^2^(1) = 62.16	<0.001
Pathological fracture	94 (3.1%)	68 (5.4)	26 (1.4)	*χ* ^2^(1) = 39.66	<0.001
Vascular injury	48 (1.57%)	10 (0.8)	38 (2.1)	*χ* ^2^(1) = 8.12	0.004
Neurological injury	63 (2.1%)	12 (1)	48 (2.6)	*χ* ^2^(1) = 11.10	0.001

**Table 4:** Bivariate association between patients' age and associated injuries with injury mechanism.

	Falls from height	Gunshot	Industrial	RTA	Simple falls	Test statistic	*p*-value
Age (years), mean (SD)	32.24 (17.99)	30.1 (10.75)	40.5 (13.63)	32.25 (14.82)	46.62 (29.36)	f (4, 134.31) = 76.2	<0.001

Age groups (years)							
Children (≤9)	55 (10.8)	2 (6.7)	0	22 (5.7)	399 (19)	*χ* ^2^(28) = 983.9	<0.001
Adolescent (10–18)	88 (17.4)	0	3 (7.9)	33 (8.5)	197 (9.4)		
Adults	358 (70.6)	28 (93.3)	35 (92.1)	323 (83)	867 (41.2)		
19–30	102 (20.1)	16 (53.3)	6 (15.8)	145 (37.3)	130 (6.2)		
31–40	95 (18.7)	9 (30)	8 (21.1)	100 (25.7)	143 (6.8)		
41–50	73 (14.4)	2 (6.7)	13 (34.2)	53 (13.6)	135 (6.4)		
51–60	58 (11.4)	1 (3.3)	5 (13.2)	21 (5.4)	207 (9.8)		
61–70	30 (5.9)	0	3 (7.9)	4 (1)	252 (12)		
Elderly (≥71)	6 (1.2)	0	0	11 (2.8)	639 (30.4)		

Associated injuries						*χ* ^2^(2) = 5.62	<0.001
Open fracture	25 (4.9)	27 (90)	37 (97.4)	56 (14.4)	38 (1.8)	*χ* ^2^(4) = 457.6	<0.001
Pathological fracture	0	0	0	0	94 (4.5)	*χ* ^2^(4) = 457.6	<0.001
Vascular injury	4 (0.8)	2 (6.7)	7 (18.4)	5 (1.3)	30 (1.4)	*χ* ^2^(4) = 28.60	<0.001
Neurological injury	13 (2.6)	3 (10)	7 (18.4)	22 (5.7)	15 (0.7)	*χ* ^2^(4) = 66.85	<0.001

Dexterity						*χ* ^2^(1) = 0.112	<0.001
Left	172 (33.9)	19 (63.3)	21 (55.3)	101 (26)	993 (47.2)	*χ* ^2^(8) = 281.9	<0.001
Right	148 (29.2)	11 (36.7)	17 (44.7)	123 (31.6)	824 (39.2)		
Axial	187 (36.9)	0	0	165 (42.4)	285 (13.6)		

^*∗*^Numbers within brackets in age group and dexterity categories represent the percentages within the same category.							
^∗∗^Numbers within brackets in the associated injury category represent the overall percentage within the injury mechanism.							

**Table 5:** Bivariate association between bone fractures with injury mechanism.

	Falls from height	Gunshot	Industrial	RTA	Simple falls	Test statistic	*p*-value
Upper limb	133 (26.2)	11 (36.7)	29 (76.3)	83 (21.3)	768 (36.5)	*χ* ^2^(12) = 311.23	<0.001

Scapula	6 (1.2)	0	0	10 (2.6)	1 (0.01)	*χ*2(4) = 35.10	<0.001

Clavicle	11 (2.2)	0	0	14 (3.6)	9 (0.4)	*χ* ^2^(4) = 31.01	<0.001

Humerus							
Proximal	9 (1.8)	1 (3.3)	0	8 (2.1)	68 (3.2)	*χ* ^2(^4) = 6.70	0.153
Shaft	5 (1)	0	0	13 (3.3)	23 (1.1)	*χ* ^2^(4) = 11.7	0.019
Distal	41 (8.1)	0	0	6 (1.5)	341 (16.2)	*χ* ^2^(4) = 118.17	<0.001

Forearm							
Proximal	8 (1.6)	0	0	5 (1.3)	34 (1.6)	*χ* ^2^(4) = 2.37	0.668
Shaft	17 (3.4)	3 (10)	1 (2.6)	10 (2.6)	92 (4.4)	*χ* ^2^(4) = 5.90	0.205
Distal	31 (6.1)	0	0	8 (2.1)	133 (6.3)	*χ* ^2^(4) = 22.16	<0.001

Hand							
Carpal	2 (0.4)	0	4 (10.5)	2 (0.5)	8 (0.4)	*χ* ^2^(4) = 18.21	0.001
Metacarpal	2 (0.4)	4 (13.3)	4 (10.5)	3 (0.8)	15 (0.7)	*χ* ^2^(4) = 30.11	<0.001
Phalangeal	1 (0.2)	3 (10)	20 (52.6)	4 (1)	44 (2.1)	*χ* ^2^(4) = 124.1	<0.001

Lower limb	185 (36.5)	19 (63.3)	9 (23.7)	137 (35.2)	1041 (49.5)	*χ* ^2^(12) = 311.23	<0.001

Femur							
Proximal	15 (3)	0	0	7 (1.8)	535 (15.4)	*χ* ^2^(4) = 239.11	<0.001
Shaft	16 (3.2)	2 (6.7)	0	28 (7.2)	72 (3.4)	*χ* ^2^(4) = 14.73	<0.001
Distal	4 (0.8)	2 (6.7)	0	7 (1.8)	52 (2.5)	*χ* ^2^(4) = 10.64	0.031

Patellar	3 (0.6)	0	0	4 (1)	18 (0.9)	*χ* ^2^(4) = 1.70	0.79

Leg							
Proximal	23 (4.5)	4 (13.3)	1 (2.6)	13 (3.3)	35 (1.7)	*χ*2(4) = 22.16	<0.001
Shaft	19 (3.7)	3 (10)	5 (13.2)	24 (6.2)	51 (2.4)	*χ*2(4) = 23.40	<0.001
Distal	41 (8.1)	4 (13.3)	0	38 (9.8)	221 (10.5)	*χ* ^2^(4) = 11.14	0.025

Foot							
Tarsal	53 (10.5)	1 (3.3)	1 (2.6)	14 (3.6)	25 (1.2)	*χ* ^2^(4) = 90.73	<0.001
Metatarsal	11 (2.2)	0	1 (2.6)	1 (0.3)	20 (1)	*χ* ^2^(4) = 9.51	0.049
Phalangeal	0	3 (10)	1 (2.6)	1 (0.3)	12 (0.6)	*χ* ^2^(4) = 19.95	0.001

Spine	141 (27.8)	0	0	111 (28.5)	246 (11.7)	*χ* ^2^(12) = 311.23	<0.001
Cervical	1 (0.2)	0	0	8 (2.1)	0	*χ* ^2^(4) = 30.50	<0.001
Thoracic	51 (10.1)	0	0	38 (9.8)	85 (4)	*χ* ^2^(4) = 44.61	<0.001
Lumbar	89 (17.6)	0	0	65 (16.7)	161 (7.7)	*χ* ^2^(4) = 71.30	<0.001

Pelvis	48 (9.5)	0	0	58 (14.9)	58 (14.9)	*χ* ^2^(4) = 120.1	<0.001

**Table 6:** Bivariate analysis between genders and age groups according to low- and high-energy injuries, *n* = 3066.

	Males				Females			
	Low energy	High energy	Test statistic	*p*-value	Low energy	High energy	Test statistic	*p*-value
Age (years), mean (SD)	38.18 (28.56)	32.92 (15.43)	*t* (1810) = 4.52	<0.001	55.48 (27.21)	31.18 (19.5)	t(1252) = 12.77	<0.001

Age groups								
Children (≤9)	257 (23.9)	53 (7.2)	*χ* ^2^(7) = 423.70	<0.001	142 (13.8)	26 (11.4)	*χ* ^2^(7) = 315.4	<0.001
Adolescent (10–18)	148 (13.8)	71 (9.6)			49 (4.8)	53 (23.2)		
Adults	437 (40.6)	601 (81.7)			432 (42.1)	143 (62.7)		
19–30 years	106 (9.9)	220 (29.9)			24 (2.3)	49 (21.5)		
31–40 years	94 (8.7)	187 (25.4)			49 (4.8)	25 (11)		
41–50 years	78 (7.2)	112 (15.2)			57 (5.6)	29 (12.7)		
51–60 years	86 (8)	59 (8)			121 (11.8)	26 (11.4)		
61–70 years	71 (6.6)	23 (3.1)			181 (17.6)	14 (6.1)		
Elderly (≥71)	236 (21.9)	11 (1.5)			403 (39.3)	6 (2.6)		

Associated injuries								
Open fracture	29 (2.7)	130 (17.7)	*χ* ^2^(1) = 122.32	<0.001	9 (0.9)	15 (6.6)	*χ* ^2^(1) = 29.34	<0.001
Pathological fracture	26 (2.4)	0	*χ* ^2^(1) = 18.04	<0.001	68 (6.6)	0	*χ* ^2^(1) = 15.98	<0.001
Vascular injury	20 (1.9)	18 (2.4)	*χ* ^2^(1) = 0.733	0.392	10 (1)	0	*χ* ^2(^1) = 1.18	0.278
Neurological injury	9 (0.8)	39 (5.3)	*χ* ^2^(1) = 33.75	<0.001	6 (0.6)	6 (2.6)	*χ* ^2^(1) = 6.23	0.013

**Table 7:** Bivariate analysis between both genders' bone fractures in low- and high-energy injuries, *n* = 3066.

	Males				Females			
	Low energy	High energy	Test statistic	*p*-value	Low energy	High energy	Test statistic	*p*-value
Upper limb	492 (45.7)	214 (29.1)	*χ* ^2^(3) = 197.60	<0.001	276 (26.9)	42 (18.4)	*χ* ^2^(3) = 71.31	<0.001

Scapula	1 (0.1)	14 (1.9)	*χ* ^2^(1) = 17.43	<0.001	0	2 (0.9)	*χ* ^2^(1) = 4.35	0.037

Clavicle	5 (0.5)	23 (3.1)	*χ* ^2^(1) = 20.30	<0.001	4 (0.4)	2 (0.9)	*χ* ^2^(1) = 0.93	0.335

Humerus								
Proximal	28 (2.6)	11 (1.5)	*χ* ^2^(1) = 2.55	0.111	40 (3.9)	7 (3.1)	*χ* ^2^(1) = 0.355	0.551
Shaft	12 (1.1)	17 (2.3)	*χ* ^2^(1) = 3.96	0.047	11 (1.1)	1 (0.4)	*χ* ^2^(1) = 0.30	0.608
Distal	214 (19.9)	33 (4.5)	*χ* ^2^(1) = 88.10	<0.001	127 (12.4)	14 (6.1)	*χ* ^2^(1) = 7.27	0.007

Forearm								
Proximal	19 (1.8)	12 (1.6)	*χ* ^2^(1) = 0.050	0.827	15 (1.5)	1 (0.4)	*χ* ^2^(1) = 0.845	0.358
Shaft	75 (7)	24 (3.3)	*χ* ^2^(1) = 11.64	0.001	17 (1.7)	7 (3.1)	*χ* ^2^(1) = 1303	0.254
Distal	82 (7.6)	34 (4.6)	*χ* ^2^(1) = 6.57	0.01	51 (5)	5 (2.2)	*χ* ^2^(1) = 3.37	0.066

Hand								
Carpal	7 (0.7)	7 (1)	*χ* ^2^(1) = 0.520	0.473	1 (0.1)	1 (0.4)	*χ* ^2^(1) = 0.100	0.802
Metacarpal	11 (1)	11 (1.5)	*χ* ^2^(1) = 0.813	0.367	4 (0.4)	2 (0.9)	*χ* ^2^(1) = 0.19	0.664
Phalangeal	38 (3.5)	28 (3.8)	*χ* ^2^(1) = 0.09	0.761	6 (0.6)	0	*χ* ^2^(1) = 0.40	0.531

Lower limb	487 (45.3)	264 (35.9)	*χ* ^2^(3) = 197.60	<0.001	554 (54)	86 (37.7)	*χ* ^2^(3) = 71.31	<0.001

Femur								
Proximal	238 (22.1)	16 (2.2)	*χ* ^2^(1) = 144.30	<0.001	297 (28.9)	6 (2.6)	*χ* ^2^(1) = 70.5	<0.001
Shaft	36 (3.3)	37 (5)	*χ* ^2^(1) = 3.20	0.074	36 (3.5)	9 (3.9)	*χ* ^2^(1) = 0.104	0.747
Distal	14 (1.3)	11 (1.5)	*χ* ^2^(1) = 0.120	0.729	38 (3.7)	2 (0.9)	*χ* ^2^(1) = 4.82	0.028

Patella	5 (0.5)	6 (0.8)	*χ* ^2^(1) = 0.890	0.345	13 (1.3)	1 (0.4)	*χ* ^2^(1) = 0.531	0.466

Leg								
Proximal	21 (2)	34 (4.6)	*χ* ^2^(1) = 10.57	0.001	14 (1.4)	7 (3.1)	*χ* ^2^(1) = 2.34	0.126
Shaft	35 (3.3)	40 (5.4)	*χ* ^2^(1) = 5.24	0.022	16 (1.6)	11 (4.8)	*χ* ^2^(1) = 7.95	0.005
Distal	99 (9.2)	58 (7.9)	*χ* ^2^(1) = 0.963	0.327	122 (11.9)	25 (11)	*χ* ^2^(1) = 0.155	0.694

Foot								
Tarsal	17 (1.6)	51 (6.9)	*χ* ^2^(1) = 34.63	<0.001	8 (0.8)	18 (7.9)	*χ* ^2^(1) = 43.1	<0.001
Metatarsal	13 (1.2)	8 (1.1)	*χ* ^2^(1) = 0.100	0.813	7 (0.7)	5 (2.2)	*χ* ^2^(1) = 3.04	0.081
Phalangeal	9 (0.8)	3 (0.4)	*χ* ^2^(1) = 1.22	0.269	3 (0.3)	2 (0.9)	*χ* ^2^(1) = 0.50	0.492

Spine	81 (7.5)	179 (24.3)	*χ* ^2^(1) = 75.22	<0.001	165 (16.1)	73 (32)	*χ* ^2^(1) = 32.90	<0.001
Cervical	0	8 (1.1)	*χ* ^2^(1) = 9.41	0.002	0	1	*χ* ^2^(1) = 0.68	0.409
Thoracic	22 (2)	58 (7.9)	*χ* ^2^(1) = 35.30	<0.001	63 (6.1)	31 (13.6)	*χ* ^2^(1) = 14.96	<0.001
Lumbar	59 (5.5)	113 (15.4)	*χ* ^2^(1) = 49.57	<0.001	102 (9.9)	41 (18)	*χ* ^2^(1) = 11.94	0.001

Pelvis	16 (1.5)	79 (10.7)	*χ* ^2^(1) = 75.22	<0.001	31 (3)	27 (11.8)	*χ* ^2^(1) = 32.90	<0.001

**Table 8:** Joint dislocation distribution, *n* = 47 patients.

	Frequency	Percentage
Hip dislocation	19	40.4
Ankle dislocation	10	21.3
Elbow dislocation	8	17
Shoulder dislocation	5	10.6
Perilunate dislocation	4	8.5
Acromioclavicular dislocation	1	2.1

## Data Availability

The data used to support the study are available upon request.

## References

[1] CDC (2022). Injury Prevention. https://www.cdc.gov/injury/wisqars/index.html.

[2] WHO (2009). *Global Status Report on Road Safety 2009*.

[3] WHO (2010). *Violence, Injuries, and Disability Biennial Report: 2008-2009*.

[4] Ryb G. E., Dischinger P. C., Kufera J. A., Burch C. A (2007). Delta V, principal direction of force, and restraint use contributions to motor vehicle crash mortality. *The Journal of Trauma, Injury, Infection, and Critical Care*.

[5] Hettrich C. M., Browner B. (2012). High-energy trauma. *Best Practice & Research Clinical Rheumatology*.

[6] Frankel V. H., Kaplan D. J., Egol K. A. (2016). Biomechanics of fractures. *Journal of Orthopaedic Trauma*.

[7] Nordin M., Frankel V. H. (2012). *Basic Biomechanics of the Musculoskeletal System*.

[8] Beaupied H., Lespessailles E., Benhamou C. L. (2007). Evaluation of macrostructural bone biomechanics. *Joint Bone Spine*.

[9] Stewart R. M. (2007). Trauma system and prevention summary for injury prevention. *The Journal of Trauma, Injury, Infection, and Critical Care*.

[10] Carter D. R., Spengler D. M. (1982). Biomechanics of fracture. *Bone in Clinical Orthopedics*.

[11] Nogueira L., Quatrehomme G., Bertrand M. F. (2017). Comparison of macroscopic and microscopic (stereomicroscopy and scanning electron microscopy) features of bone lesions due to hatchet hacking trauma. *International Journal of Legal Medicine*.

[12] Young L., Rule G. T., Bocchieri R. T., Walilko T. J., Burns J. M., Ling G. (2015). When physics meets biology: low and high-velocity penetration, blunt impact, and blast injuries to the brain. *Frontiers in Neurology*.

[13] Love J. C. (2019). Sharp force trauma analysis in bone and cartilage: a literature review. *Forensic Science International*.

[14] Crowder C., Rainwater C. W., Fridie J. S. (2013). Microscopic analysis of sharp force trauma in bone and cartilage: a validation study. *Journal of Forensic Sciences*.

[15] Fadl S. A., Sandstrom C. K. (2019). Pattern recognition: a mechanism-based approach to injury detection after motor vehicle collisions. *RadioGraphics*.

[16] Pastakia K., Kumar S. (2011). Acute whiplash associated disorders (WAD). *Open Access Emergency Medicine: OAEM*.

[17] Bragg K. J., Varacallo M. (2022). Cervical sprain. *StatPearls [Internet]. Treasure Island (FL)*.

[18] Panday P., Vikram A., Chawla A., Mukherjee S. (2021). Prediction of lower extremity injuries in car-pedestrian crashes—real-world accident study. *Traffic Injury Prevention*.

[19] Chong S. L., Chiang L. W., Allen J. C., Fleegler E. W., Lee L. K. (2018). Epidemiology of pedestrian-motor vehicle fatalities and injuries, 2006-2015. *American Journal of Preventive Medicine*.

[20] Feng X. Y., Nah S. A., Lee Y. T., Lin Y. C., Chiang L. W. (2015). Pedestrian injuries in children: who is most at risk?. *Singapore Medical Journal*.

[21] Kannus P., Sievänen H., Palvanen M., Järvinen T., Parkkari J. (2005). Prevention of falls and consequent injuries in elderly people. *The Lancet*.

[22] Moreland B., Kakara R., Henry A. (2020). Trends in nonfatal falls and fall-related injuries among adults aged ≥ 65 Years—United States, 2012-2018. *MMWR Morb Mortal Wkly Rep*.

[23] Tuckel P., Milczarski W., Silverman D. G. (2018). Injuries caused by falls from playground equipment in the United States. *Clinical Pediatrics*.

[24] McAndrew M. P., Johnson K. D. (1991). Penetrating orthopedic injuries. *Surgical Clinics of North America*.

[25] Bartlett C. S., Helfet D. L., Hausman M. R., Strauss E. (2000). Ballistics and gunshot wounds: effects on musculoskeletal tissues. *Journal of the American Academy of Orthopaedic Surgeons*.

[26] Stewart T. C., Grant K., Singh R., Girotti M (2004). Pediatric trauma in southwestern ontario: linking data with injury prevention initiatives. *The Journal of Trauma, Injury, Infection, and Critical Care*.

[27] Roudsari B. S., Shadman M., Ghodsi M. (2006). Childhood trauma fatality and resource allocation in injury control programs in a developing country. *BMC Public Health*.

[28] Elkbuli A., Dowd B., Spano P. J., McKenney M. (2020). Pediatric seat belt use in motor vehicle collisions: the need for driver education programs. *Journal of Trauma Nursing*.

[29] Bergh C., Wennergren D., Möller M., Brisby H. (2020). Fracture incidence in adults in relation to age and gender: a study of 27, 169 fractures in the Swedish Fracture Register in a well-defined catchment area. *PLoS One*.

[30] Meena R. K., Singh A. M., Singh C. A., Chishti S., Kumar A. G., Langshong R. (2013). Pattern of fractures and dislocations in a tertiary hospital in North-East India. *The Internet Journal of Epidemiology*.

[31] World Health Organization (2010). *Global Health Observatory (Gho)*.

[32] Airaksinen N. K., Handolin L. E., Heinänen M. T. (2020). Severe traffic injuries in the Helsinki trauma Registry between 2009-2018. *Injury*.

[33] Mansuri F. A., Al-Zalabani A. H., Zalat M. M., Qabshawi R. I. (2015). Road safety and road traffic accidents in Saudi Arabia. A systematic review of existing evidence. *Saudi Medical Journal*.

[34] Rovere G., Perna A., Meccariello L. (2021). Epidemiology and aetiology of male and female sexual dysfunctions related to pelvic ring injuries: a systematic review. *International Orthopaedics*.

[35] Bledsoe L., Alessi K., Toro J. B., Giordano B., Hanypsiak B. T. (2018). Fragility fractures: diagnosis and treatment. *American Journal of Orthopedics*.

[36] Kanis J. A. (2002). Diagnosis of osteoporosis and assessment of fracture risk. *The Lancet*.

[37] Nabian M. H., Zadegan S. A., Zanjani L. O., Mehrpour S. R. (2017). Epidemiology of joint dislocations and ligamentous/tendinous injuries among 2, 700 patients: five-year trend of a tertiary center in Iran. *Arch Bone Jt Surg*.

[38] AlRousan F. M., Alkhawaldah A., Altarawneh R. Y., Qudah A., Almigdad A. K. (2022). Impact of social distancing on geriatric hip fractures among Jordanian population during COVID-19 pandemic. *Mater Sociomed*.

